# Serum neurofilament light protein predicts clinical outcome in traumatic brain injury

**DOI:** 10.1038/srep36791

**Published:** 2016-11-07

**Authors:** Pashtun Shahim, Magnus Gren, Victor Liman, Ulf Andreasson, Niklas Norgren, Yelverton Tegner, Niklas Mattsson, Niels Andreasen, Martin Öst, Henrik Zetterberg, Bengt Nellgård, Kaj Blennow

**Affiliations:** 1 Clinical Neurochemistry Laboratory, Institute of Neuroscience and Physiology, Sahlgrenska Academy at University of Gothenburg, Sahlgrenska University Hospital, Mölndal, SE-43180 Mölndal, Sweden; 2 UmanDiagnostics, Umeå, Sweden; 3 Division of Medical Sciences, Department of Health Sciences, Luleå University of Technology, SE 971 87 Luleå, Sweden; 4 Clinical Memory Research Unit, Lund University, Malmö, Sweden; 5 Department of NVS, Karolinska Institute, Center for Alzheimer Research, Stockholm, Sweden; 6 Department of Anaesthesiology and Intensive Care, Institute of Clinical Sciences, Sahlgrenska Academy, Gothenburg University, Gothenburg, Sweden; 7 Department of Molecular Neuroscience, UCL Institute of Neurology, London WC1N1PJ, UK

## Abstract

Axonal white matter injury is believed to be a major determinant of adverse outcomes following traumatic brain injury (TBI). We hypothesized that measurement of neurofilament light protein (NF-L), a protein found in long white-matter axons, in blood samples, may serve as a suitable biomarker for neuronal damage in TBI patients. To test our hypotheses, we designed a study in two parts: i) we developed an immunoassay based on Single molecule array technology for quantification of NF-L in blood, and ii) in a proof-of-concept study, we tested our newly developed method on serial serum samples from severe TBI (sTBI) patients (n = 72) and controls (n = 35). We also compared the diagnostic and prognostic utility of NF-L with the established blood biomarker S100B. NF-L levels were markedly increased in sTBI patients compared with controls. NF-L at admission yielded an AUC of 0.99 to detect TBI versus controls (AUC 0.96 for S100B), and increased to 1.00 at day 12 (0.65 for S100B). Importantly, initial NF-L levels predicted poor 12-month clinical outcome. In contrast, S100B was not related to outcome. Taken together, our data suggests that measurement of serum NF-L may be useful to assess the severity of neuronal injury following sTBI.

Traumatic brain injury (TBI) is the most common cause of death and disability following blunt trauma among young people worldwide^1^. In clinical setting, severity of TBI is commonly classified using Glasgow Coma Scale (GCS), where severe TBI (sTBI) has a GCS score of 3–8. Structural damage following sTBI may be detected by advanced magnetic resonance (MRI) techniques, but is not alone sufficient to predict long-term clinical outcome^2^,^3^. In addition, clinical variables such as age, GCS, pupil reactivity and the extent or grade of damage on imaging have shown some promise in predicting outcome following TBI, however, with limitation^4^.

Axonal white matter injury has been hypothesized to be the primary determinant of outcome following both mild and severe TBI^5^. Neurofilament light (NF-L) is a CNS-enriched protein, abundantly expressed in the long myelinated subcortical white matter axons^6^. Together with the neurofilament medium (NF-M) and heavy (NF-H) subunits, NF-L is one of the scaffolding proteins of the neural cytoskeleton, with important roles in axonal and dendritic branching and growth^7^. In the context of TBI, measurement NF-L in cerebrospinal fluid (CSF) has shown prognostic utility, both for mild and sTBI^8^. However, owing to the invasive nature of lumbar puncture for accessing CSF, it may not always be practical to perform repeated lumbar punctures on a routine clinical basis in TBI cases, and thus blood-based biomarkers are more preferable. The current most commonly used blood biomarkers for brain injury, S100 calcium binding protein B (S100B) and neuron-specific enolase (NSE), where the former is used in the emergency setting instead of CT scan to rule out mild TBI have limited diagnostic and prognostic value^9^,^10^,^11^.

A major challenge of developing blood-based tests has been the lack of highly sensitive immunochemical methods for detection of CNS-specific markers in peripheral blood. We have recently measured tau, a cortical axonal protein in plasma using Single molecule array (Simoa) technology, which is up to 1000-fold more sensitive than conventional ELISA, and showed that tau measured 1 hour after concussion were significantly elevated in concussed athletes^12^. However, large myelinated axons, which are enriched in neurofilament are believed to be more vulnerable to traumatic brain injury^8^, suggesting that neurofilament measured in blood may have better both diagnostic and prognostic utility than tau for acute TBI. Previous studies have attempted to quantify NF-M, and NF-H in patients with stroke and TBI using traditional ELISA, where only less than 50% of patients with TBI had increased serum NF-M with significantly higher levels in polytrauma patients, thus casting doubt on the utility of NF-M as a specific biomarker for TBI^13^,^14^. However, of the three subunits of neurofilament, NF-L is the most abundant and essential component of the neurofilament core, acting as the backbone to which NF-M and NF-H co-assemblies^15^,^16^. Recently, a study used a commercial standard ELISA for measurement of NF-L in serum of patients with sTBI, where they found that high levels of serum NF-L may have prognostic utility for sTBI^17^. However, one of the major limitations of using traditional ELISA for quantification of low-abundance protein such as neurofilament in peripheral fluid is not being able to measure minor axonal damage in TBI cases or compare with levels found in healthy subjects.

With the above in mind, we hypothesized that measurement of NF-L in sera of humans with sTBI would be helpful to acutely assess the severity of traumatic axonal injury. To test our specific hypotheses, we designed a study in 2 parts: i) we developed an ultrasensitive ELISA based on Simoa technology^18^, for quantification of NF-L in serum, and ii) in a proof-of-concept study, we applied our newly developed assay on serial blood samples of sTBI patients (n = 72), as well as neurologically healthy controls (n = 35). We also compared the diagnostic and prognostic utility of NF-L with the established biomarker for brain injury, S100B.

## Results

### Assay development

In the first part of this study, we developed an ultrasensitive assay for quantification of NF-L in serum. For detailed description of the assay development see the method section of the supplementary material. In a subset of sTBI patients (n = 32) who underwent ventriculostomy for intracranial pressure (ICP) monitoring, NF-L was also measured in ventricular CSF (vCSF). The levels of NF-L in serum correlated (r = 0.52, p < 0.0001) with the levels of vCSF NF-L in these patients (see Supplementary Fig. 1b).

### Demographic and clinical characteristics

The clinical and demographic characteristics of the sTBI patients are summarized in Table 1. There were no significant differences in age (median, 36 years, interquartile range [IQR] 22–54 years versus median, 31 years, IQR 27–60 year; p = 0.60) and sex (p = 0.07, determined by Pearson’s chi-square test) between sTBI patients and the control group. The etiology of the trauma included assault (8%), fall (29%), motorcycle accident (26%), car accident (27%), and other cause (10%, explosion and unknown trauma). At 12 months follow-up, 10 patients had died (these patient had died during 30 days of injury), 20 patients had severe disability, 23 had moderate disability and 17 had good recovery as assessed by the GOS scale (Table 1). No patients were in a persistent vegetative state (GOS score of 2). Two of the patients who survived did not come to the 12-month follow-up and were excluded.

### S-NF-L in patients with severe TBI shows similar dynamics as in ventricular CSF

**Figure 1:**
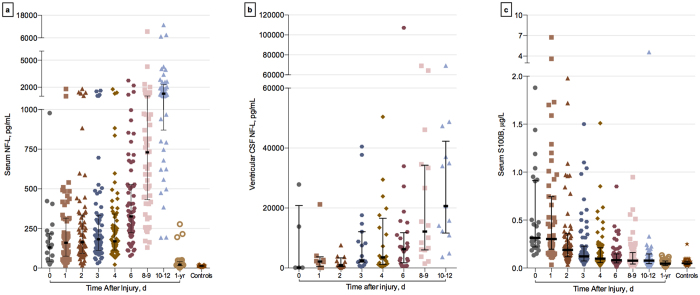
Temporal profile of the biomarkers in severe traumatic brain injury. (**a**) NF-L levels at admission and latter sampling time points (days 1–12) were increased in patients with TBI as compared to controls (***p < 0.001, all). (**b**) NF-L measured in ventricular cerebrospinal fluid from the patients who underwent ventriculostomy show similar releasing dynamics as in serum. S100B levels at day 0 (admission), day 1, day 2, day 3, day 4 (***p < 0.001, all), day 6 (*p < 0.05) were increased in sTBI patients compared with controls, and levels measured at latter time points were not statistically different from the control group (**c**). Values are presented as median; error bars indicate interquartile range.

Serum NF-L levels were significantly increased from day 0 (admission to neurointensive care unit) to day 12, and the highest levels were measured at 12 days after injury. The rise in serum NF-L between day 0 and day 12 were significant as compared to controls (p < 0.0001, all time), and the levels normalized at 1-year follow-up (Fig. 1a). Similar to the releasing dynamics observed in serum, the levels of NF-L in vCSF rose over time with the highest levels measured at 12 days after injury (Fig. 1b). In contrast, the highest levels of S100B were measured at day 1 after the injury, and the levels fell to almost normal levels during days 2–12 after injury (Fig. 1c).

### Serum NF-L separates severe TBI patients from controls

**Figure 2:**
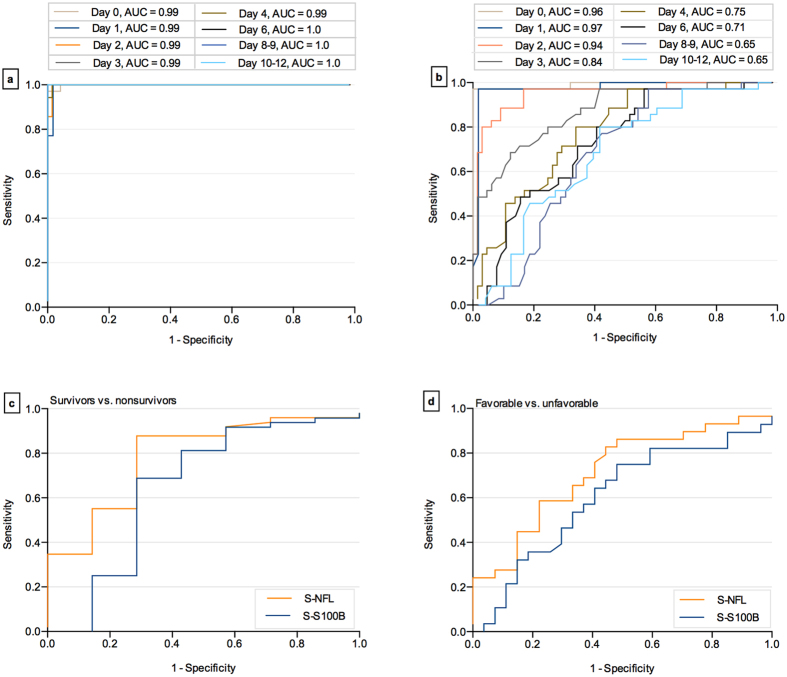
Diagnostic accuracy of the biomarkers for severe traumatic brain injury. Area under the receiver operating characteristic curve (AUC) for NF-L (**a**) and S100B (**b**) concentrations at admission, and days 1–12 post-TBI versus control group. **(c)** shows the ROC curve for NF-L and S100B in survivors versus non-survivors. **(d)** shows the ROC curve for NF-L and S100B in favorable versus unfavorable outcome group. AUC for NF-L and S100B were 0.70 and 0.65 in survivors versus non-survivors; 0.71 and 0.60 in favorable versus unfavorable outcome, respectively.

As a proof-of-concept, we assessed the diagnostic utility of NF-L for sTBI. We analyzed AUC comparing the NF-L levels tested at different times after TBI with the control group. We also assessed the diagnostic utility of S100B. NF-L at admission yielded an AUC of 0.99 versus. 0.96 for S100B, and the AUC for NF-L increased to 1.0 versus 0.65 for S100B at day 12 (Fig. 2a,b). The optimal individual cut-off level for NF-L was calculated as 24.0 pg/mL (Youden index, 0.96) and for S100B as 0.142 μg/L (Youden index, 0.93). Applying these cut-off levels yielded a sensitivity of 97%, a specificity of 96% and a positive likelihood ratio (LR+) of 23.0 for NF-L. The sensitivity, specificity and positive LR for S100B were 96%, 96% and 24.0, respectively.

### Serum NF-L correlates with clinical outcome variables

Initial levels of serum NF-L correlated with pupil reactivity, and Marshall computed tomography (CT) classification scores (r = 0.50, p < 0.001, and r = 0.38, p = 0.010, respectively; Table 1). There was no significant difference in the level of NF-L between GCS categories (p = 0.08; Table 1). Also, S100B correlated with pupil reactivity (r = 0.30, p = 0.030; Table 1). There was no significant correlation between S100B and Marshall CT classification score (r = 0.23, p = 0.12; Table 1).

The levels of either S-NFL or S100B differed between the *APOE* ε4 carriers and non-carriers (p = 0.80 and p = 0.60, respectively; Table 1).

### Serum NF-L separates survivors from non-survivors

**Figure 3:**
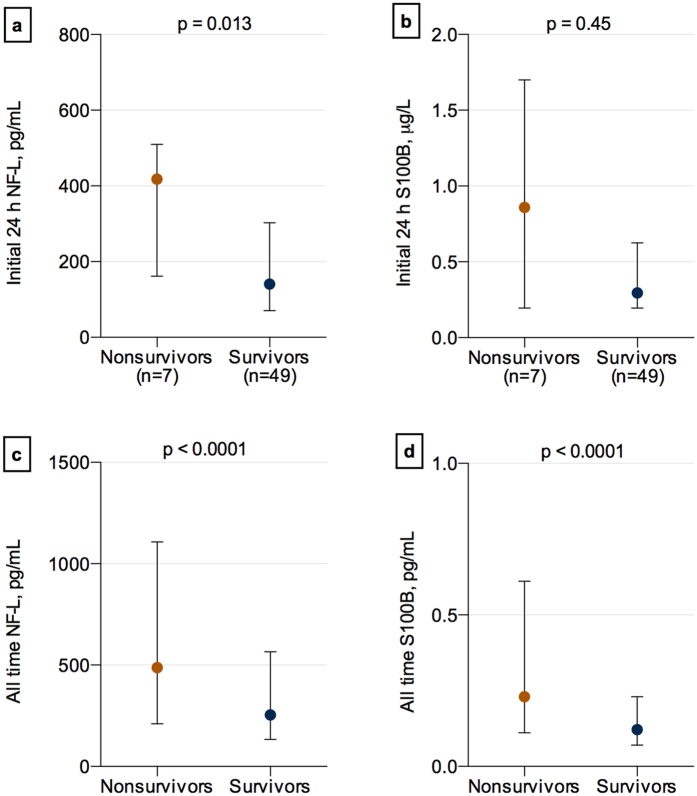
Serum NF-L and S100B in non-survivors versus survivors of severe traumatic brain injury. NF-L (**a**) and S100B (**b**) in serum samples obtained at 24 hours after injury. NF-L (**c**) and S100B (**d**) levels in serum samples obtained over the period of 1–12 days after injury (**d**). Values are presented as median; error bars indicate interquartile range.

NF-L levels at 24 hours after injury were significantly higher in non-survivors versus survivors (p = 0.0010) (Fig. 3a). Also, NF-L levels over the 12-day period were significantly higher in non-survivors as compared to survivors (Fig. 3c). In contrast, there were no significant differences in the levels of S100B at 24 hours, after sTBI between non-survivors and survivors (p = 0.45) (Fig. 3b). However, overall levels of S100B were increased in non-survivors as compared to survivors (Fig. 3d).

In addition, optimal cut-off levels for NF-L (411 pg/mL) and S100B (0.45 μg/L) were derived from the Youden index for predicting survival. NF-L measured at admission could discriminate between survivors and nonsurvivors with a sensitivity, specificity, and LR+ of 71%, 88%, and 6.0, respectively. Sensitivity, specificity, and LR+ for S100B were 69%, 71%, and 2.4, respectively (Fig. 2c). In addition, we performed multivariate analysis with classification trees, where NF-L measured at 24 hours could independently predict outcome (AUC = 1.0) versus S100B (AUC = 0.96). Both of the biomarkers increased the AUC to 1.0 when added to the full model with clinical outcome variables as suggested by the international mission for prognosis and clinical trial (IMPACT) studies.

### Serum NF-L correlates with GOS score at 12-months follow-up

**Figure 4:**
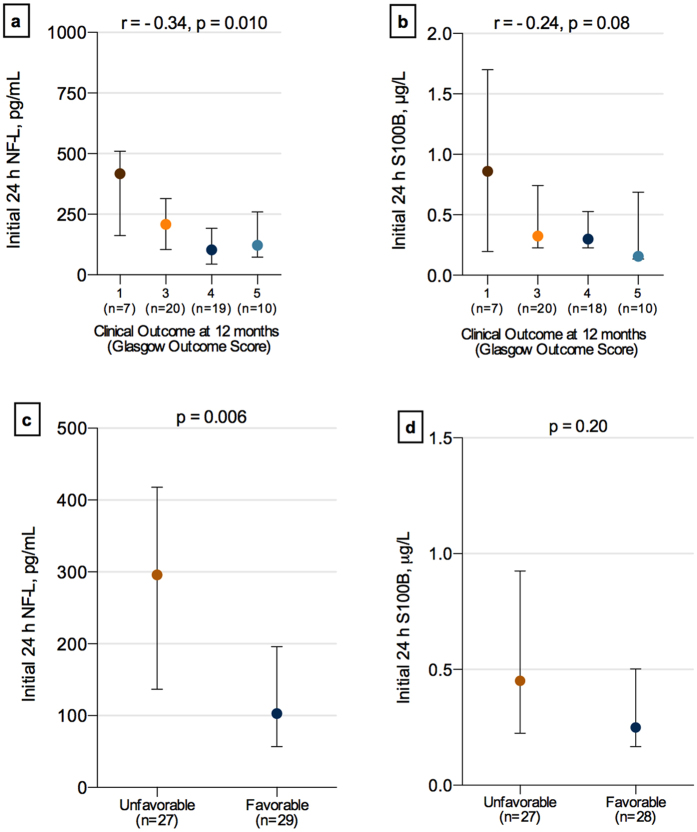
NF-L is predictive of overall clinical outcome 12 months after injury in severe traumatic brain injury. Serum NF-L samples (**a**) obtained at initial 24 hours after injury significantly differed between the Glasgow Outcome Scale (GOS) categories (GOS 1 versus. GOS 2–5; *** p < 0.0001). (**b**) There was no significant difference in the levels of S100B and different GOS categories when corrected for multiple group comparisons. No patients were in a persistent vegetative state (GOS score of 2). (**c**) Serum NF-L levels during the initial 24 hours of sampling were significantly higher in patients with unfavorable outcome versus favorable. (**d**) There was no significant difference in the levels of S100B between patients with unfavorable versus favorable outcome. Values are presented as median; error bars indicate interquartile range.

We assessed the univariate relationship between early (24 hours) NF-L levels and clinical outcome, and found higher levels of NF-L in patients with lower GOS score at 12 months after injury (r = − 0.34, p = 0.010) (Fig. 4b). In contrast, there was no significant difference in the levels of S100B between GOS categories (Fig. 4b). However, there were trends toward higher levels in patients with poor outcome (Fig. 4b). Also, there was a borderline significant relationship between early levels of S100B and GOS score (r = − 0.24, p = 0.08).

Traditionally, outcome following sTBI may also be dichotomized into favorable (GOS 1–3) and unfavorable (GOS 4–5). The levels of NF-L at 24 hours after injury were significantly higher in the unfavorable group as compared to the favorable group (Fig. 4c). NF-L (216 pg/mL, Youden index, 0.38) at 24 hours of sampling could separate patients with favorable versus unfavorable outcome with a sensitivity, specificity, and LR+ of 83%, 56%, and 1.9 compared with S100B (0.44 μg/L, Youden index, 0.27) that had 75%, 55% and 1.6, respectively (Fig. 2d). In addition, in the multivariate analysis with classification trees, NF-L levels at 24 hours increased the possibility to predict outcome (AUC = 0.70) in a prediction model including also age, pupil, GCS, and APOE, compared to the model without NF-L (AUC 0.65). In contrast, S100B did not improve the prediction model (AUC 0.65) compared to the model without S100B (AUC 0.65).

## Discussion

In this study, we developed an ultrasensitive ELISA for quantification of the axonal white matter protein NF-L in serum using the Simoa platform. This assay allows for quantification of NF-L in blood samples down to 2.7 pg/mL, with a limit of detection of 0.27 pg/mL, enabling measurement in all samples, including samples from healthy subjects. In short, we found increased serum levels of both NF-L and S100B in patients with sTBI compared with controls, but the dynamics were different; whilst NF-L concentrations were increased at admission to NICU, and continued to rise over the first 12 days post-injury, S100B concentrations fell over time after an initial peak the first day after trauma. Importantly, initial NF-L levels correlated with clinical outcome, whereas S100B showed a weaker association.

The finding that NF-L increases in serum of TBI patients is in agreement with two previous studies on NF-L measured in CSF of amateur boxers with concussive and sub-concussive head trauma^8^,^19^. In addition, a recent study applying the commercial CSF ELISA method for measurement of NF-L in serum samples also found an increase in serum NF-L in sTBI patients^17^. However, the commercial ELISA method developed for NF-L measurement in CSF samples^17^ is approximately 100 times less sensitive than the Simoa assay presented in this paper. The lower limit of detection was reported to be 31 ng/L^17^, which is in contrast to the analytical sensitivity of 78 ng/L for the same commercial assay found in another paper^20^. This means that samples with normal or mildly elevated levels will escape detection, which precludes identification of a cut-off value for normality and hinders accurate measurement of serum NF-L mild TBI cases. Indeed, in a study directly comparing these methods, the correlations between NFL levels measured in paired CSF and serum samples were much stronger for the Simoa method (r = 0.88) than for the commercial ELISA (r = 0.38), mainly due to the inability of the ELISA to measure lower levels^20^.

An intriguing observation arising from our study is that NF-L and S100B showed different dynamics; with NF-L rising in a linear fashion over time, while S100B returning to approximately normal levels after 2–12 days after injury. It is possible that serum NF-L may increase beyond the 12^th^ day sampling time-point, but we were unable to monitor this in the present study since no samples were available after this time-point (except for the year 1 sample). Animal model studies indicate that the half-life for NF-L in mice optical axons and retinal ganglion neurons may be in the vicinity of three weeks^21^, but there are no human studies that have determined the half-life of NF-L either in serum or CSF. Furthermore, NF-L measured in vCSF of sTBI patients displayed similar releasing dynamics to the NF-L measured in serum, and also a strong correlation was observed between NF-L measured in lumbar CSF and serum NF-L (r = 0.81) from the same individuals in this study, suggestive of neuronal release. The stronger correlation between lumbar CSF NF-L and serum NF-L versus vCSF NF-L and serum NF-L might be due to the gradient difference; much higher concentrations of NF-L in vCSF compared to the lumbar. Importantly, the dynamic of NF-L observed in this study also fits well with the implication that all axonal injury lesions may not occur or may not be visible on brain CT in the initial stages of TBI, but evolves over hours or days as the axonal swelling progresses^22^.

Secondary effects of injury such as inflammation, as well as genetic variability have previously been shown in an animal model study of TBI to effect NF-L concentration^23^. However, we found no significant difference in the levels of either NF-L or S100B between the *APOE* ε4 carriers and non-carriers. Previous studies have shown conflicting results regarding the effect of *APOE* ε4 on outcome following TBI^24^.

S100B showed similar dynamics as in previous studies with the highest concentrations within 48 hours of admittance to the NICU^25^. Previous studies of S100B in the context of sTBI, have also observed a secondary peak, where the secondary peaks were correlated to secondary injury, however, in the current study, we did not observe any obvious secondary peaks^26^,^27^,^28^. It is worth mentioning that the delta value for the secondary peak observed in the previous studies was very small compared with the S100B levels found at day 1.

Clinical outcome is highly variable after sTBI, and prognostic prediction therefore difficult to make despite association of clinical variables such as age, pupil reactivity, GCS score, and the extent of damage on imaging at admission in statistical multivariate models^4^,^29^. In this study, we found significant correlation between higher serum NF-L levels and pupil reactivity as well as Marshall CT score. Furthermore, NF-L could separate survivors from non-survivors, and the initial levels were predictive of 12 months adverse clinical outcomes. Additionally, in the multivariate analysis, initial levels of NF-L could independently predict outcome, and increased the prognostic value when added to the model with clinical outcome variables as suggested by the IMPACT studies^3^. Similar to NF-L, initial levels of S100B correlated with pupil reactivity but not Marshall CT score. Also, S100B marginally correlated with GOS score. Additionally, S100B in multivariate analyses could independently separate survivors from the non-survivors, while it showed poorer predictive value than NF-L for prediction of GOS score. Previous studies on S100B have also showed conflicting results for predicting outcome^12^,^30^,^31^,^32^,^33^.

Developing a reliable method for quantification of axonal white matter injury in peripheral fluid may have utility beyond the field of TBI. CSF NF-L has also been shown to increase in axonal degenerative disease such as Alzheimer’s disease (AD), multiple sclerosis (MS) and amyotrophic lateral sclerosis (ALS) and frontotemporal dementia (FTD)^8^,^34^,^35^,^36^,^37^,^38^. Thus, measurement of NF-L in serum may have utility in the clinical and research settings for these axonal degenerative diseases, where currently biomarker-supported diagnosis and are limited to analyses of CSF.

There are limitations to this study. First, we could not directly assess the relationship of NF-L concentrations and axonal injury evaluated by MRI techniques, such as diffusion tensor imaging (DTI). A previous study did not find a correlation between serum NF-L and diffuse axonal injury on DTI, which may be due the limited number of subjects in the DTI group, or since no anti-HAMA reagents were included in the ELISA method used^17^, possibly also from interference from heterophilic antibodies that may result in very high levels. In contrast, a recent study using the same ultrasensitive Simoa assay as in the present paper, showed a marked (30-fold) increase in serum NFL in patients with sTBI, with higher NFL levels showing a tight correlation with MR-DTI measures of DAI^39^. Further, a recent study on CSF NF-L in demyelinating disorders point toward NF-L being a marker of axonal injury, with the concentration of NF-L correlating with the extent of axonal injury on MRI^40^. Also, we could not assess the relationship of NF-L to vCSF closely in all patients, as we did not have access to vCSF from all patients. Additionally, we compared the diagnostic utility of NF-L with healthy controls. It is possible that multitrauma patients without head injury may also have increased level of NF-L, although, we observed no significant effect of physical contact or trauma on serum levels of NF-L in ice hockey players after a game without head injury, thus, speaking against NF-L being expressed in extracerebral tissues (see Supplementary Table 1). In contrast, there was a significant increase in the level of S100B after the game without head injury, suggestive of extracerebral release (see Supplementary Table 1).

In conclusion, we developed an ultrasensitive ELISA that allowed for the quantification of NF-L in serum of individuals with sTBI as well as healthy controls. Our data add support for the hypothesis that axonal white injury may be related to long-term impairment following TBI. Measurement of NF-L levels in serum may be useful way to assess the severity of axonal injury acutely in the intensive care unit, as well as in athletes with concussion. Additionally, the newly developed ultrasensitive assay for NF-L could be useful in both clinical and research settings to track disease progression, and also evaluate efficacy of early phase clinical pharmacodynamics testing of candidate therapeutics, not only in TBI, but also other axonal degenerative diseases such as MS and FTD, where biomarker-supported diagnosis and prognosis have previously been limited to analyses of CSF.

## Methods

### Standard protocol approvals and consent

The ethics committee for medical research at the University of Gothenburg, Sweden approved the use of human subjects for these studies. All the methods in the study were performed in accordance with the relevant guidelines and regulations of the ethics committee for medical research at the University of Gothenburg. Written informed consent was obtained from all participants or, if incapable, from their proxy, prior to the inclusion in the study.

### Participants

For the sTBI part of this study, 72 patients were recruited consecutively at Sahlgrenska University Hospital, Sweden, over a period of 11 months. Samples from two of the patients were excluded from the analyses due to loss of follow-up at 12 months. The inclusion criteria were: 1) sTBI with a GCS of 8 or less at admission, 2) admittance to the NICU within 48 hours from head injury, 3) age ≥18 years, 4) acceptance from next-of-kin to participate in the study, and 5) residence in Sweden for 12 months follow-up. The exclusion criteria were no informed consent, known history of neurological and/or autoimmune disease, and pregnancy. Blood samples were obtained at admission (n = 25) and day 1 (n = 56), 2 (n = 68), 3 (n = 62), 4 (n = 64), 6 (n = 66), 8–9 (n = 57), and 10–12 days (n = 50) post-trauma and at 12 months (n = 32) clinical follow-up. In the sTBI patients (n = 32), who underwent insertion of an ICP monitor using ventriculostomy, we also measured NF-L in vCSF. The control group consisted of 35 neurologically healthy subjects with normal mini-mental state examination scores and no history of head trauma or other potential causes of brain injury.

### Clinical outcome assessment

In patients with sTBI, the primary outcome was Glasgow Outcome Scale (GOS) score at 12 months follow-up. GOS is quantified into scores of 1 to 5, and can be divided into either unfavorable (death, vegetative state, severe disability, GOS 1–3) or favorable (moderate disability, good recovery, GOS 4–5) outcome^41^,^42^. We also assessed the association of serum NF-L and S100B levels with other clinical predictors of outcome, suggested by the IMPACT studies^3^,^4^, such as age at injury, CT Marshall classification scores, GCS score, and pupil reactivity^43^. The Marshall classification describes whether the injury is diffuse with different degrees of severity or focal^44^. We also tested whether the presence of *APOE* ε4 in sTBI hade effect on biomarker levels or outcome as some studies have reported that *APOE* ε4 may be associated with adverse clinical outcomes after sTBI^45^.

### Biochemical procedures

Blood samples were collected by venipuncture into gel-separator tubes for serum and centrifuged within 20–60 minutes. Serum was separated, aliquoted and stored at −80 °C pending biochemical analysis.

vCSF NF-L levels were measured using a commercial ELISA (NF-light^®^ ELISA, Uman Diagnostics, Umeå, Sweden) as described previously^46^.

S100B levels were measured using Cobas e601 (Roche Diagnostics, Mannheim, Germany) with the commercially available Elecsys S100 test (Roche Diagnostics, Mannheim, Germany, lower limit of detection < 0.005 μg/L, see Methods in the supplementary material for detailed description of the method.

NF-L levels in serum were measured using the Simoa platform (Quanterix, Lexington, MA, USA), a magnetic bead-based digital ELISA that allows detection of proteins at subfemtomolar concentrations. Limit of detection (LOD) for the NF-L assay was 0.29 pg/mL and lower limit of quantification (LLOQ) was 2.7 pg/mL when compensated for a four-fold sample dilution. LOD and LLOQ were determined by mean blank signal +3 SD and +10 SD, respectively. Average intra-assay duplicate coefficient of variation (CV) for the samples was 6.5% (SD 8.6%). Details on the development of NF-L assay can be found in the Methods of the Supplement.

All samples were analyzed using the same batch of reagents by board-certified laboratory technicians who were blind to clinical information.

### Statistical analyses

The area under the receiver operating characteristic curve was calculated for determining diagnostic accuracy. The Youden index was applied for optimal individual cut-off levels. Outcome was also assessed using recursive partitioning analysis regression of trees using the “rpart” package^47^. All statistical analyses were performed using GraphPad Prism 5.0 (GraphPad Inc., San Diego, CA), and R (v. 3.0.3, The R Foundation for Statistical Computing). Details on the statistical methods applied in this study can be found in the Statistics of the Supplement.

## Additional Information

**How to cite this article**: Shahim, P. *et al*. Serum neurofilament light protein predicts clinical outcome in traumatic brain injury. *Sci. Rep.* **6**, 36791; doi: 10.1038/srep36791 (2016).

**Publisher’s note**: Springer Nature remains neutral with regard to jurisdictional claims in published maps and institutional affiliations.

## Supplementary Material

Supplementary Information

## Figures and Tables

**Table 1:** Association of clinical outcome variable and biomarker levels.

Variables	NF-L, pg/mL, Median (IQR)	S100B, μg/L, Median (IQR)
GCS score		
GCS 6–8, n = 45	196 (89–413), p = 0.08	0.30 (0.20–0.9), p = 0.15
GCS 3–5, n = 25	107 (67–190)	0.24 (0.18–0.32)
Pupil reactivity		
Both reactive, n = 31	90 (54–177), r = 0.50, p < 0.0001	0.23 (0.16–0.31), r = 0.30, p = 0.030
One reactive, n = 20	266 (122–450)	0.60 (0.26–1.0)
Neither reactive, n = *16*	314 (120–490)	0.30 (0.14–1.5)
Missing, n *=*3	NA	NA
Marshall CT classification		
Grade I, n = 0	0, r = 0.38, p = 0.001	0, r = 0.23, p = 0.12
Grade II, n = 18	132 (66–376)	0.23 (0.13–0.25)
Grade III, n = 15	141 (61–203)	0.30 (0.16–0.40)
Grade VI, n = 28	212 (98–413)	0.45 (0.25–1.0)
Missing, n = 9	NA	NA
GOS scale at 1-yr		
GOS 1, death, n = 10	418 (162–510), p = 0.014	0.86 (0.20–1.7), p = 0.08
GOS 2, vegetative state, n = 0	0	0
GOS 3, severe disability, n = 20	210 (105–315)	0.32 (0.23–0.74)
GOS 4, moderate disability, n = 23	103 (45–192)	0.30 (0.20–0.53)
GOS 5, good recovery, n = 17	121 (74–259)	0.15 (0.13–0.70)
*APOE* ε4+, n = 17	160 (72–314), p = 0.80	0.31 (0.2–0.9), p = 0.60
*APOE* ε4−, n = 53	166 (80–356)	0.28 (0.18–0.6)
Controls, n = 35	13 (11–17), p < 0.0001	0.05 (0.04–0.07), p < 0.0001

Abbreviations: sTBI, severe traumatic brain injury; GCS, Glasgow coma scale; GOS, Glasgow outcome score; NF-L, Neurofilament light protein; S100B, S100 calcium-binding B. NA denotes not applicable/availabe. *APOE* ε4+ genotype was defined by the presence of at least one *APOE ε4* allele. GOS was assesses at 12 months follow-up. P-values are for associations with each variable and biomarker levels (continuous), tested by Spearman rank correlation (pupil reactivity, Marshall CT classification, and GOS scale) or Mann-Whitney *U* test (GCS score, APOE genotype and controls).

## References

[b1] Murray C. J. & Lopez A. D. Global mortality, disability, and the contribution of risk factors: Global Burden of Disease Study. Lancet 349, 1436–1442, doi: 10.1016/S0140-6736(96)07495-8 (1997). 9164317

[b2] Collaborators M. C. T. . Predicting outcome after traumatic brain injury: practical prognostic models based on large cohort of international patients. Bmj 336, 425–429, doi: 10.1136/bmj.39461.643438.25 (2008). 18270239 PMC2249681

[b3] Murray G. D. . Multivariable prognostic analysis in traumatic brain injury: results from the IMPACT study. Journal of neurotrauma 24, 329–337, doi: 10.1089/neu.2006.0035 (2007). 17375997

[b4] Steyerberg E. W. . Predicting outcome after traumatic brain injury: development and international validation of prognostic scores based on admission characteristics. PLoS medicine 5, e165 discussion e165, doi: 10.1371/journal.pmed.0050165 (2008). 18684008 PMC2494563

[b5] Smith D. H., Meaney D. F. & Shull W. H. Diffuse axonal injury in head trauma. The Journal of head trauma rehabilitation 18, 307–316 (2003). 16222127 10.1097/00001199-200307000-00003

[b6] Zetterberg H., Smith D. H. & Blennow K. Biomarkers of mild traumatic brain injury in cerebrospinal fluid and blood. Nature reviews. Neurology 9, 201–210, doi: 10.1038/nrneurol.2013.9 (2013). 23399646 PMC4513656

[b7] Lepinoux-Chambaud C. & Eyer J. Review on intermediate filaments of the nervous system and their pathological alterations. Histochemistry and cell biology 140, 13–22, doi: 10.1007/s00418-013-1101-1 (2013). 23749407

[b8] Zetterberg H. . Neurochemical aftermath of amateur boxing. Archives of neurology 63, 1277–1280, doi: 10.1001/archneur.63.9.1277 (2006). 16966505

[b9] Routsi C. . Increased levels of serum S100B protein in critically ill patients without brain injury. Shock 26, 20–24, doi: 10.1097/01.shk.0000209546.06801.d7 (2006). 16783193

[b10] Stalnacke B. M., Ohlsson A., Tegner Y. & Sojka P. Serum concentrations of two biochemical markers of brain tissue damage S-100B and neurone specific enolase are increased in elite female soccer players after a competitive game. British journal of sports medicine 40, 313–316, doi: 10.1136/bjsm.2005.021584 (2006). 16556784 PMC2577522

[b11] Hasselblatt M. . Serum S100beta increases in marathon runners reflect extracranial release rather than glial damage. Neurology 62, 1634–1636 (2004). 15136701 10.1212/01.wnl.0000123092.97047.b1

[b12] Shahim P. . Blood biomarkers for brain injury in concussed professional ice hockey players. JAMA neurology 71, 684–692, doi: 10.1001/jamaneurol.2014.367 (2014). 24627036

[b13] Martinez-Morillo E. . Neurofilament medium polypeptide (NFM) protein concentration is increased in CSF and serum samples from patients with brain injury. Clinical chemistry and laboratory medicine: CCLM/FESCC 53, 1575–1584, doi: 10.1515/cclm-2014-0908 (2015). 25720124

[b14] Gatson J. W. . Detection of neurofilament-H in serum as a diagnostic tool to predict injury severity in patients who have suffered mild traumatic brain injury. Journal of neurosurgery 121, 1232–1238, doi: 10.3171/2014.7.JNS132474 (2014). 25192482

[b15] Heins S. . The rod domain of NF-L determines neurofilament architecture, whereas the end domains specify filament assembly and network formation. The Journal of cell biology 123, 1517–1533 (1993). 8253847 10.1083/jcb.123.6.1517 PMC2290863

[b16] Lee M. K., Xu Z., Wong P. C. & Cleveland D. W. Neurofilaments are obligate heteropolymers *in vivo*. The Journal of cell biology 122, 1337–1350 (1993). 8376466 10.1083/jcb.122.6.1337 PMC2119859

[b17] Al Nimer F. . Comparative Assessment of the Prognostic Value of Biomarkers in Traumatic Brain Injury Reveals an Independent Role for Serum Levels of Neurofilament Light. PloS one 10, e0132177, doi: 10.1371/journal.pone.0132177 (2015). 26136237 PMC4489843

[b18] Rissin D. M. . Single-molecule enzyme-linked immunosorbent assay detects serum proteins at subfemtomolar concentrations. Nature biotechnology 28, 595–599, doi: 10.1038/nbt.1641 (2010). PMC2919230 20495550

[b19] Neselius S. . CSF-biomarkers in Olympic boxing: diagnosis and effects of repetitive head trauma. PloS one 7, e33606, doi: 10.1371/journal.pone.0033606 (2012). 22496755 PMC3319096

[b20] Kuhle J. . Comparison of three analytical platforms for quantification of the neurofilament light chain in blood samples: ELISA, electrochemiluminescence immunoassay and Simoa. Clinical chemistry and laboratory medicine: CCLM/FESCC, doi: 10.1515/cclm-2015–1195 (2016). 27071153

[b21] Barry D. M., Millecamps S., Julien J. P. & Garcia M. L. New movements in neurofilament transport, turnover and disease. Experimental cell research 313, 2110–2120, doi: 10.1016/j.yexcr.2007.03.011 (2007). 17451679

[b22] Narayan R. K. . Clinical trials in head injury. Journal of neurotrauma 19, 503–557, doi: 10.1089/089771502753754037 (2002). 12042091 PMC1462953

[b23] Al Nimer F. . Strain influences on inflammatory pathway activation, cell infiltration and complement cascade after traumatic brain injury in the rat. Brain, behavior, and immunity 27, 109–122, doi: 10.1016/j.bbi.2012.10.002 (2013). 23044177

[b24] Zhou W. . Meta-analysis of APOE4 allele and outcome after traumatic brain injury. Journal of neurotrauma 25, 279–290, doi: 10.1089/neu.2007.0489 (2008). 18373478

[b25] Ercole A., Thelin E. P., Holst A., Bellander B. M. & Nelson D. W. Kinetic modelling of serum S100b after traumatic brain injury. BMC neurology 16, 93, doi: 10.1186/s12883-016-0614-3 (2016). 27315805 PMC4912776

[b26] Thelin E. P., Nelson D. W. & Bellander B. M. Secondary peaks of S100B in serum relate to subsequent radiological pathology in traumatic brain injury. Neurocritical care 20, 217–229, doi: 10.1007/s12028-013-9916-0 (2014). 24146416

[b27] Berger R. P., Bazaco M. C., Wagner A. K., Kochanek P. M. & Fabio A. Trajectory analysis of serum biomarker concentrations facilitates outcome prediction after pediatric traumatic and hypoxemic brain injury. Developmental neuroscience 32, 396–405, doi: 10.1159/000316803 (2010). 20847541 PMC3215242

[b28] Raabe A. . S-100B protein as a serum marker of secondary neurological complications in neurocritical care patients. Neurological research 26, 440–445, doi: 10.1179/016164104225015958 (2004). 15198874

[b29] Lingsma H. F., Roozenbeek B., Steyerberg E. W., Murray G. D. & Maas A. I. Early prognosis in traumatic brain injury: from prophecies to predictions. Lancet neurology 9, 543–554, doi: 10.1016/S1474-4422(10)70065-X (2010). 20398861

[b30] Metting Z., Wilczak N., Rodiger L. A., Schaaf J. M. & van der Naalt J. GFAP and S100B in the acute phase of mild traumatic brain injury. Neurology 78, 1428–1433, doi: 10.1212/WNL.0b013e318253d5c7 (2012). 22517109

[b31] Ryb G. E. . S-100beta does not predict outcome after mild traumatic brain injury. Brain injury: [BI] 28, 1430–1435, doi: 10.3109/02699052.2014.919525 (2014). 24911665

[b32] Korfias S. . Serum S-100B protein monitoring in patients with severe traumatic brain injury. Intensive care medicine 33, 255–260, doi: 10.1007/s00134-006-0463-4 (2007). 17143637

[b33] Vos P. E. . GFAP and S100B are biomarkers of traumatic brain injury: an observational cohort study. Neurology 75, 1786–1793, doi: 10.1212/WNL.0b013e3181fd62d2 (2010). 21079180

[b34] Magnoni S. . Tau elevations in the brain extracellular space correlate with reduced amyloid-beta levels and predict adverse clinical outcomes after severe traumatic brain injury. Brain: a journal of neurology 135, 1268–1280, doi: 10.1093/brain/awr286 (2012). 22116192 PMC3326246

[b35] Skillback T. . CSF neurofilament light differs in neurodegenerative diseases and predicts severity and survival. Neurology 83, 1945–1953, doi: 10.1212/WNL.0000000000001015 (2014). 25339208

[b36] Tortorella C. . Cerebrospinal fluid neurofilament tracks fMRI correlates of attention at the first attack of multiple sclerosis. Multiple sclerosis 21, 396–401, doi: 10.1177/1352458514546789 (2015). 25168208

[b37] Lu C. H. . Neurofilament light chain: A prognostic biomarker in amyotrophic lateral sclerosis. Neurology, doi: 10.1212/WNL.0000000000001642 (2015). PMC4456658 25934855

[b38] Zetterberg H. . Association of Cerebrospinal Fluid Neurofilament Light Concentration With Alzheimer Disease Progression. JAMA neurology 73, 60–67, doi: 10.1001/jamaneurol.2015.3037 (2016). 26524180 PMC5624219

[b39] Ljungqvist J., Zetterberg H., Mitsis M., Blennow K. & Skoglund T. Serum neurofilament light protein as a marker for diffuse axonal injury - results from a case series study. Journal of neurotrauma In press (2016). 10.1089/neu.2016.4496 27539721

[b40] Eikelenboom M. J. . Multiple sclerosis: Neurofilament light chain antibodies are correlated to cerebral atrophy. Neurology 60, 219–223 (2003). 12552034 10.1212/01.wnl.0000041496.58127.e3

[b41] Wilson J. T., Pettigrew L. E. & Teasdale G. M. Structured interviews for the Glasgow Outcome Scale and the extended Glasgow Outcome Scale: guidelines for their use. Journal of neurotrauma 15, 573–585 (1998). 9726257 10.1089/neu.1998.15.573

[b42] Jennett B. & Bond M. Assessment of outcome after severe brain damage. Lancet 1, 480–484 (1975). 46957 10.1016/s0140-6736(75)92830-5

[b43] Isoniemi H., Tenovuo O., Portin R., Himanen L. & Kairisto V. Outcome of traumatic brain injury after three decades--relationship to ApoE genotype. Journal of neurotrauma 23, 1600–1608, doi: 10.1089/neu.2006.23.1600 (2006). 17115907

[b44] Maas A. I., Hukkelhoven C. W., Marshall L. F. & Steyerberg E. W. Prediction of outcome in traumatic brain injury with computed tomographic characteristics: a comparison between the computed tomographic classification and combinations of computed tomographic predictors. Neurosurgery 57, 1173-1182 discussion 1173–1182 (2005). 16331165 10.1227/01.neu.0000186013.63046.6b

[b45] Blennow K., Hardy J. & Zetterberg H. The neuropathology and neurobiology of traumatic brain injury. Neuron 76, 886–899, doi: 10.1016/j.neuron.2012.11.021 (2012). 23217738

[b46] Norgren N., Rosengren L. & Stigbrand T. Elevated neurofilament levels in neurological diseases. Brain research 987, 25–31 (2003). 14499942 10.1016/s0006-8993(03)03219-0

[b47] Breiman L., Friedman, J., Olshen R. A. & Stone C. I. Classification and Regression Trees. (Boca Raton:: FL CRC Press, , 1984).

