# Supplementary material for: Serum neurofilament light protein predicts clinical outcome in traumatic brain injury
Source: Sci Rep. 2016 Nov 7;6:36791. doi: 10.1038/srep36791 (PMC5098187; doi:10.1038/srep36791)
Supplement: Supplementary Information [file srep36791-s1.doc]

**Title: Serum neurofilament light protein predicts clinical outcome in traumatic brain injury**

**Authors:** Pashtun Shahim^1^, MD, PhD, Magnus Gren^1^, MD, Victor Liman^1^, BSc, Ulf Andreasson^1^, PhD, Niklas Norgren^2^, PhD, Yelverton Tegner^3^, MD, PhD, Niklas Mattsson^4^, MD, PhD, Niels Andreasen^5^, MD, PhD, Martin Öst^6^, MD, PhD, Henrik Zetterberg^1,7^, MD, PhD, Bengt Nellgård^6^, MD, PhD, Kaj Blennow^1^, MD, PhD

**Affiliations:**

^1^Clinical Neurochemistry Laboratory, Institute of Neuroscience and Physiology, Sahlgrenska Academy at University of Gothenburg, Sahlgrenska University Hospital, Mölndal, SE-43180 Mölndal, Sweden

^2^UmanDiagnostics, Umeå, Sweden

^3^Division of Medical Sciences, Department of Health Sciences, Luleå University of Technology, SE 971 87 Luleå, Sweden

^4^Clinical Memory Research Unit, Lund University, Malmö, Sweden

^5^Department of NVS, Karolinska Institute, Center for Alzheimer Research, Stockholm, Sweden

^6^Department of Anaesthesiology and Intensive Care, Institute of Clinical Sciences, Sahlgrenska Academy, Gothenburg University, Gothenburg, Sweden

^7^Department of Molecular Neuroscience, UCL Institute of Neurology, London WC1N1PJ, UK

**Correspondence:** Kaj Blennow, MD, PhD

Clinical Neurochemistry Lab

Dept. of Neuroscience and Physiology

University of Gothenburg, Mölndal Hospital

Sahlgrenska University Hospital

SE-43180 Mölndal, Sweden

Tel (mobile): +46 (0) 76 107 38 35

Fax: +46 (0) 31 41 92 89

E-mail: [kaj.blennow@neuro.gu.se](mailto:pashtun.shahim@neuro.gu.se)

*Supplementary Data:*

Methods, detailed description of the assay measuring S100B in serum

Methods, detailed description of the assay measuring NF-L in serum

Assay development

Figure 1, Verification of the accuracy of digital ELISA method

Statistics, detailed descriptions of the statistics

Table 1, NF-L and S100B prior and after a game of ice hockey

**Supplementary Data**

*Methods, measurement of S100B in serum*

Serum S100B levels were measured using Cobas e601 (Roche Diagnostics, Mannheim, Germany) with the commercially available Elecsys S100 test (Roche Diagnostics, Mannheim, Germany, lower limit of detection < 0.005 µg/L). The Cobas e601 is a fully automated immunoassay using electrochemiluminescence for detection. Samples were incubated with two monoclonal antibodies; one biotinylated and one labelled with ruthenium. Streptavidin-coated magnetic particles were added to bind the sandwich complexes. The particles were magnetically attracted to the measuring cell where a signal was produced, deriving from excitement of the ruthenium molecules and their subsequent emission of light. Concentrations were determined using a curve stored in the instrument, which was calibrated prior to analysis. Samples were run undiluted with single determinations. Quality controls (PreciControl Universal, Roche Diagnostics, Mannheim, Germany) were run prior to the samples and fulfilled the acceptance criteria set by the manufacturer.

*Methods, measurement of NF-L in serum*

NF-L levels in serum were measured using the Simoa platform (Quanterix, Lexington, MA, USA), a magnetic bead-based digital ELISA that allows detection of proteins at subfemtomolar concentrations. Magnetic beads (Quanterix, Lexington, MA, USA) were conjugated with capture antibody UD1 (UmanDiagnostics Art No. 37005, Umeå, Sweden) at 0.3 mg/mL according to bead supplier’s conjugation protocol. Prior to each run, serum samples were diluted 10 fold, NF-L calibrator (UmanDiagnostics Art No. 27001, Umeå, Sweden) was series diluted and biotin-labeled detection antibody UD2 (UmanDiagnostics Art No. 37017, Umeå, Sweden) was diluted to 0.1 µg/mL in PBS, 0.1 % Tween20, 2 % BSA, 10 µg/mL TRU Block (Meridian Life Science, Inc., Memphis, TN, USA). For each determination, 400 000 conjugated beads were washed and resuspended in 100 μL serum sample or calibrator and 20 µL detection antibody was added. After a 30 minute incubation, beads were washed and resuspended in 100 µL streptavidin-conjugated β-galactosidase (Quanterix, Lexington, MA, USA) at 150 pM diluted in SBG Diluent (Quanterix, Lexington, MA, USA). Following five minutes of incubation, the beads were washed and transferred together with resorufin-D-galactopyranoside substrate (Quanterix, Lexington, MA, USA) to an array of wells, each well only big enough to contain one bead. The array was imaged with a charge-coupled device (CCD) camera imaging system and the images were used to differentiate between empty beads and beads with bound analyte, giving a signal expressed as average enzyme per bead (AEB). To extract concentrations from AEBs, each sample AEB was fitted to a four-parameter logistic curve plotted from the known concentrations of the NF-L calibrator run in parallel with the samples. Calibrator points were run in triplicates while samples were run in duplicates. All samples from each individual patient were measured within the same run. Limit of detection (LOD) for the NF-L assay was 0.29 pg/mL and lower limit of quantification (LLOQ) was 2.7 pg/mL when compensated for a four-fold sample dilution. LOD and LLOQ were determined by mean blank signal + 3 SD and + 10 SD, respectively. Average intra-assay duplicate coefficient of variation (CV) for the samples was 6.5 % (SD 8.6 %).

*Assay development*

The pilot version of the assay was initially tested on 68 healthy subjects (median age 27 years; range 19-40 years). We noticed abnormally increased levels of NF-L in four of these. After thorough evaluation of the assay, we discovered that the four subjects with abnormally high NF-L had heterophilic antibodies (HA). The effect of HA was neutralized by adding anti-HA reagent/blocker, which removed the interference.

The modified assay was then verified on sera samples from 18 individuals with known quantities of CSF NF-L (Fig. 1a). Further, in a subset of TBI patients (n = 32) who underwent ventriculostomy for ICP monitoring, NF-L was also measured in vCSF. The levels of NF-L in serum were related (r = 0.52, p < 0.0001) to the levels of vCSF NF-L in these patients (Fig.1b).

**Figure 1. Verification of the accuracy of digital ELISA method.**

(a) Relationship between serum NF-L and CSF NF-L. (b) Relationship between serum NF-L and ventricular CSF from the same individuals.

*Methods, statistics*

We used Pearson χ^2^ test to test for differences in categorical variables between TBI vs. control group. For the group comparison of NF-L levels at 1 day after the injury versus the control group, the Mann-Whitney *U* test or Students t-test was used, when appropriate, and for comparison of biomarker levels between sTBI and controls at all measured time points, the Kruskal-Wallis test by ranks was used. Dunn’s correction was performed for all multiple comparisons. The relationship between biomarker levels and at admission and age were tested with Spearman’s rank correlation, as well as GOS scale score at 12 months.

The area under the receiver operating characteristic curve (AUC) was calculated for determining the diagnostic accuracy at admission and 1-12 day post-TBI vs. the control group. Biomarker accuracies were calculated as sensitivity, specificity, and LR+. The sensitivity was the proportion that tested positive among all true positives, the specificity was the proportion that tested negative among all true negatives and the LR+ was calculated as sensitivity/(1−specificity). The Youden index was applied for calculating optimal (*i.e.*, maximum Youden index) individual cut-off levels for NF-L and S100B. The Youden index for a cut-off is defined by its sensitivity + specificity – 1.

We performed recursive partitioning analysis regression trees using the “rpart” package for predicting outcome (Breiman L et al. 1984). In our predictive model, binary variables (favourable vs. unfavourable outcome or survivor vs. non-survivors) were as dependent variable, and biomarkers at 24 hours, age, GCS score, pupil reactivity and *APOE* ε4 as independent predictors. We tested the models with and without the biomarkers, in order to estimate whether the biomarkers increased the prediction of the clinical outcome variables. In order to avoid overestimation due to overfitting, the decision tree properties were evaluated using 10-folds cross-validated area under the receiver operating characteristic curve.

All tests were two-sided and statistical significance was determined at *P*< 0.05 (two-sided). All statistical analyses were performed using R (v. 3.0.3, The R Foundation for Statistical Computing), and GraphPad Prism 5.0 (GraphPad Inc., San Diego, CA).

| **Table 1. Demographic data and serum concentrations of the biomarkers prior and after a game.** | | | |
| --- | --- | --- | --- |
| **Characteristics** | **Prior**  **(n = 26)** | **1 h**  **(n = 20)** | **p value**^†^ |
| Age − year | 28.0 (26-31) | 27.5 (21-31) | 0.92 |
| NF-L, pg/mL | 10.8 (9.0-14.0) | 10.6 (9.0-15.0) | 0.80 |
| S100B, μg/L | 0.04 (0.03-0.06) | 0.06 (0.04-0.07) | 0.025 |
| Abbreviations: NF-L, neurofilament light protein; S100B, S100 calcium-binding B  Age and biomarker concentrations are median (IQR).  ^†^P values are for the comparison between the baseline (1 hour prior to the game) and  1 hour after the game. | | | |
